# Out-of-Focus Projector Calibration Method with Distortion Correction on the Projection Plane in the Structured Light Three-Dimensional Measurement System

**DOI:** 10.3390/s17122963

**Published:** 2017-12-20

**Authors:** Jiarui Zhang, Yingjie Zhang, Bo Chen

**Affiliations:** School of Mechanical Engineering, Xi’an Jiaotong University, Xi’an 710049, Shaanxi, China; yjzhang@mail.xjtu.edu.cn (Y.Z.); chenbo156@stu.xjtu.edu.cn (B.C.)

**Keywords:** calibration, out-of-focus projector, distortion correction, focal plane, projection plane

## Abstract

The three-dimensional measurement system with a binary defocusing technique is widely applied in diverse fields. The measurement accuracy is mainly determined by out-of-focus projector calibration accuracy. In this paper, a high-precision out-of-focus projector calibration method that is based on distortion correction on the projection plane and nonlinear optimization algorithm is proposed. To this end, the paper experimentally presents the principle that the projector has noticeable distortions outside its focus plane. In terms of this principle, the proposed method uses a high-order radial and tangential lens distortion representation on the projection plane to correct the calibration residuals caused by projection distortion. The final accuracy parameters of out-of-focus projector were obtained using a nonlinear optimization algorithm with good initial values, which were provided by coarsely calibrating the parameters of the out-of-focus projector on the focal and projection planes. Finally, the experimental results demonstrated that the proposed method can accuracy calibrate an out-of-focus projector, regardless of the amount of defocusing.

## 1. Introduction

**Figure 1:**
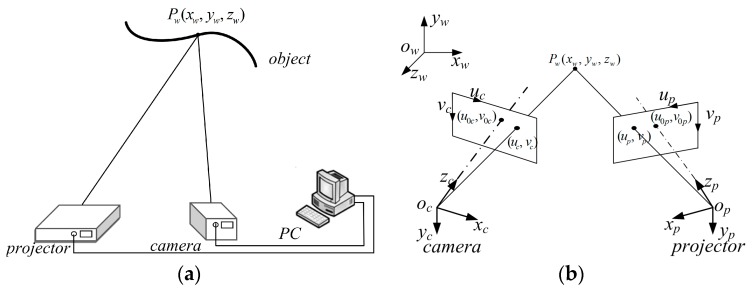
The principle of the structured light three-dimensional (3D) measurement system: (**a**) system components; and, (**b**) measurement principle.

Optic three-dimensional (3D) shape measurement has been wildly studied and applied due to its speed, accuracy, and flexibility. Examples of such applications include industrial inspection, reverse engineering, and medical diagnosis, etc. [1,2]. As shown in Figure 1, a typical structured light measurement system is composed by a camera and a projector. The projector projects a series of encoding fringe patterns onto the surface of object, and the camera captures the distorted patterns caused by the depth variation of the surface. Finally, the 3D surface point is reconstructed based on triangulation, providing that the system parameters have been obtained through the system calibration. Hence, in this system, one of the crucial aspects is to accurately calibrate the camera and projector, as the measurement accuracy is ultimately influenced by the calibration accuracy.

Camera calibration has been extensively studied, and a variety of camera calibration approaches have been proposed. Several traditional camera calibration methods exist, including direct linear transformation, nonlinear camera calibration method, two-steps camera calibration method, self-calibration, and Zhang’s method [3,4,5,6,7]. Moreover, some advanced camera calibration methods that are based on traditional methods have been proposed [8,9,10,11]. Qi et al. proposed a method that applies the stochastic parallel gradient descent (SPGD) algorithm to resolve the frequent iteration and long calibration time deficiencies of the traditional two-step camera calibration method [8]. Kamel et al. used three more objective functions to speed up the convergence rate of the nonlinear optimization in the two-step camera calibration method [9]. Huang et al. improved the calibration accuracy of the camera by using an active phase target and statistically constrained bundle adjustment (SCBA) [10]. Jia et al. proposed an improved camera calibration method, based on perpendicularity to eliminate the effect of non-perpendicularity of the camera motion on calibration accuracy in the binocular stereo vision measurement system [11]. However, the projector calibration is more complicated, since it is a programmable light source without a capturing function. The existing projector calibration methods can be classified into two categories: phase-to-depth model and inverse camera method. For the phase-to-depth model, projector calibration occurs by establishing the relationship between the depth and the phase value [12,13,14]. However, the fitting polynomial is always complicated. The inverse camera method is widely used for its small number of calibration parameters and low-cost to compute. In this method, the projector is usually treated as a device with inverse optics of the camera, and thus the camera calibration method can be applied to the projector calibration. This paper used the inverse camera model to calibrate the projector. With this method, the object world coordinates of the projection points are calculated by the calibrated camera, and then the projection points are used to calibrate the projector using the same approach as the camera calibration [15,16]. This calibration technique is simple and convenient, but errors can be significant if the camera is not well-calibrated. To solve the problem of error transmission from the camera to the projector, the virtual camera method is proposed in order to help the projector to “see” the scene. The corresponding relationship of the calibration points between the pixels on the projector Digital Micro-mirror Device (DMD) and the pixels on the camera Charge-Coupled Device (CCD) can be established. Then, the projector was calibrated using the same method as the camera, by using the corresponding points on the projector image and the calibration board [17,18,19]. This calibration approach does not depend on the accuracy of the camera calibration and can achieve higher accuracy, yet its process is complex. Additionally, some methods exist for improving the calibration accuracy of projector by improving the detection accuracy of the reference feature centers [20], or correcting the error caused by lens distortion [21,22].

Recently, the optical 3D measurement technology has been focusing on increasing the speed and accuracy of the measurement method. Lei and Zhang proposed the binary defocusing patterns projection technology, which projects defocused binary structured patterns instead of sinusoidal patterns for 3D measurement [23,24,25,26]. The binary defocusing technique has the advantages of high measuring speed, removing the errors in measurement results caused by the nonlinear gamma of the projector, and having no rigid requirement for precise synchronization [27]. However, calibrating the binary defocusing patterns projection measurement system introduces many challenges because the projector is substantially defocused. Moreover, most of the well-established accurate calibration methods for structured light systems require the projector to be in focus. For calibration with projector defocusing, two attempts have been made. Merner et al. [28] attempted to calibrate the structured light system with an out-of-focus projector, in which the pixels z were a low-order polynomial function of absolute phase, and the (x, y) coordinates were calculated from the camera calibration with a known z value. In this method, high depth accuracy was achieved, but the spatial precision was limited. Li et al. [29] analyzed the defocused imaging system model to confirm the center of the projector pixel still corresponded to the center of a camera pixel, regardless the amount of defocusing, and built the one-to-one mapping between the camera pixel and the center point of the projector pixel in the phase domain. Finally, the structured light system, with an out-of-focus projector, was calibrated by the standard OpenCV camera calibration toolbox that was based on the model described by Zhang’s method [7]. Although Li et al.’s calibration method reached an accuracy of about 73 µm for a proper calibration volume, the method neglected the influence of the amount of defocusing on the calibration parameters, which always significantly influence the measurement results.

To address the limitations of the approaches mentioned above, an improved out-of-focus projector calibration method was proposed in this paper, where a distortion correction method on the projection plane and nonlinear optimization algorithm were adopted. The proposed method was composed of two steps: coarse calibration and final calibration. The coarse calibration was to find out approximate parameters and initial values for final calibration based on nonlinear optimization algorithm. To this end, two special planes of the out-of-focus projector, the focal plane, and the projection plane, were given more attention. In the coarse calibration process, the calibration plane was moved to the focal plane (or focal plane 1) of an out-of-focus projector, and the projector was calibrated as an inverse camera by the pinhole camera model. The intrinsic and extrinsic parameters matrices on the focal plane were selected as the initial value of the final out-of-focus projector calibration. Secondly, we considered the lens distortion on the projection plane as the initial value of the final projector calibration. To calculate the lens distortion on the projection plane using the pinhole camera model, the defocusing projector should be adjusted so it is focused on the projection plane, and was it calibrated as a standard inverse camera. Finally, based on the re-projection mathematical model with distortion, the final accuracy parameters of the out-of-focus projector were obtained with a nonlinear optimization algorithm. The objective function was to minimize the sum of the re-projection error of all the reference points onto the projector image plane. In addition, the paper experimentally presents the principle that the projector has noticeable distortions outside its focus plane. When compared to the traditional calibration method, the experiment results demonstrated that our proposed method can accurately calibrate an out-of-focus projector regardless of the amount of defocusing.

This paper is organized as follows. Section 2 explains the basic principles used for the proposed calibration method. Section 3 presents calibration principle and process. Section 4 shows the experimental results to verify the performance of our calibration method, and Section 5 summarizes this paper.

## 2. Mathematical Model

### 2.1. Camera Model

**Figure 2:**
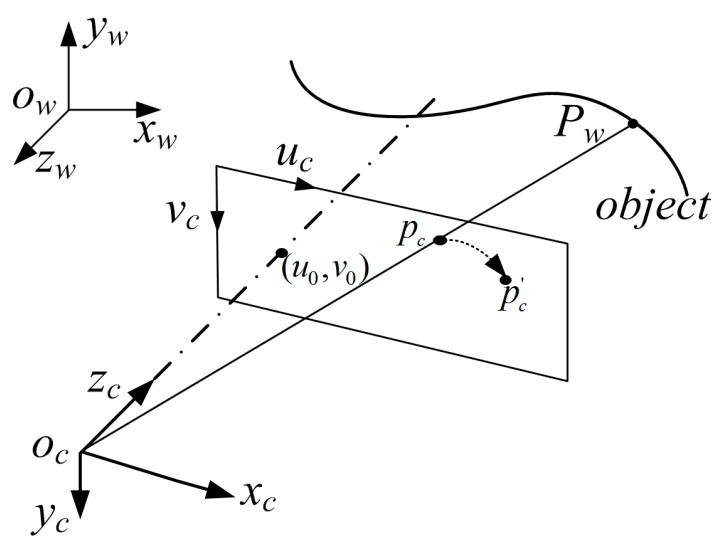
Pinhole camera model.

The well-known pinhole model is used to describe a camera with intrinsic and extrinsic parameters. The intrinsic parameters include focal length, principle point, and pixel skew factor. The rotation matrix and translation vector, which define the relationship between a world coordinate system and the camera coordinate system, are the extrinsic parameters [7]. As shown in Figure 2, a 3D point in world coordinate system $o_{w}-x_{w}y_{w}z_{w}$ can be represented by $P_{w}={\left\{x_{w},\;y_{w},\;z_{w}\right\}}^{T}$ and the corresponding two-dimensional (2D) point in camera coordinate system $o_{c}-x_{c}y_{c}z_{c}$ is $p_{c}={\left\{u_{c},\;v_{c}\right\}}^{T}$. The relationship between a 3D point $P_{w}$ and its imaging point $p_{c}$ can be described as follows:

(1) $$s{\tilde{p}}_{c}=A_{c}[R_{c},\;T_{c}]{\tilde{P}}_{w}$$

where ${\tilde{p}}_{c}={\left\{u_{c},\;v_{c},\;1\right\}}^{T}$ is the homogeneous coordinate of the point $p_{c}$ in the camera imaging coordinate system, ${\tilde{P}}_{w}={\left\{x_{w},\;y_{w},\;z_{w},\;1\right\}}^{T}$ is the homogeneous coordinate of the point $p_{w}$ in the world coordinate system,s is a scale factor, $\left[R_{c}\;,\;T_{c}\right]$ is the camera extrinsic matrices, $R_{c}$ denotes the rotation matrix, which is a 3 × 3 matrix, and $T_{c}$ is the translation vector. $A_{c}$ represents the intrinsic matrices, which can be described as follows:

(2) $$A_{c}=\left[\begin{array}{ccc} f_{x} & \gamma & u_{0}\\ 0 & f_{y} & v_{0}\\ 0 & 0 & 1 \end{array}\right]$$

where $f_{x}$ and $f_{y}$ are elements implying the focal lengths along the $u_{c}$, $v_{c}$ axes of the image plane, respectively, and γ is the skew factor of the $u_{c}$ and $v_{c}$ axes. For modern cameras, γ=0. $\left(u_{0}\;,\;v_{0}\right)$ is the coordinate of the principal point in the camera imaging plane.

Furthermore, the image coordinates in the above equations were considered without distortion. The lens distortion of the camera should be corrected to improve the calibration accuracy. Several models exist for lens distortion, such as a radial and tangential distortion model [30,31], rational function distortion model [32,33], division distortion model [31,34], and so on [35]. In this paper, a typical radial and tangential distortion model was used due to its simplicity and sufficient accuracy, formulated as:

(3) $$\{\begin{array}{c} u_{c}^{\prime }=u_{c}+\delta_{uc}=u_{c}+[k_{1}u_{r}r^{2}+k_{2}u_{r}r^{4}+p_{1}(3u_{r}^{2}+v_{r}^{2})+2p_{2}u_{r}v_{r}]\\ v_{c}^{\prime }=v_{c}+\delta_{vc}=v_{c}+[k_{1}v_{r}r^{2}+k_{2}v_{r}r^{4}+p_{2}(u_{r}^{2}+3v_{r}^{2})+2p_{1}u_{r}v_{r}] \end{array}$$

where $\left(u_{c}^{\prime },\;v_{c}^{\prime }\right)$ represents the imaging point on the imaging plane of camera with radial and tangential correction, $\left(u_{c},\;v_{c}\right)$ is the imaging point before correction, and $r=\sqrt{u_{r}{}^{2}+v_{r}{}^{2}}$ is the absolute distance between the imaging point $\left(u_{c},\;v_{c}\right)$ and the original point $\left(u_{0},\;v_{0}\right)$. $k_{1},\;k_{2}$ are the radial distortion coefficients, and $p_{1},\;p_{2}$ are the tangential coefficients.

### 2.2. Light Encoding

**Figure 3:**
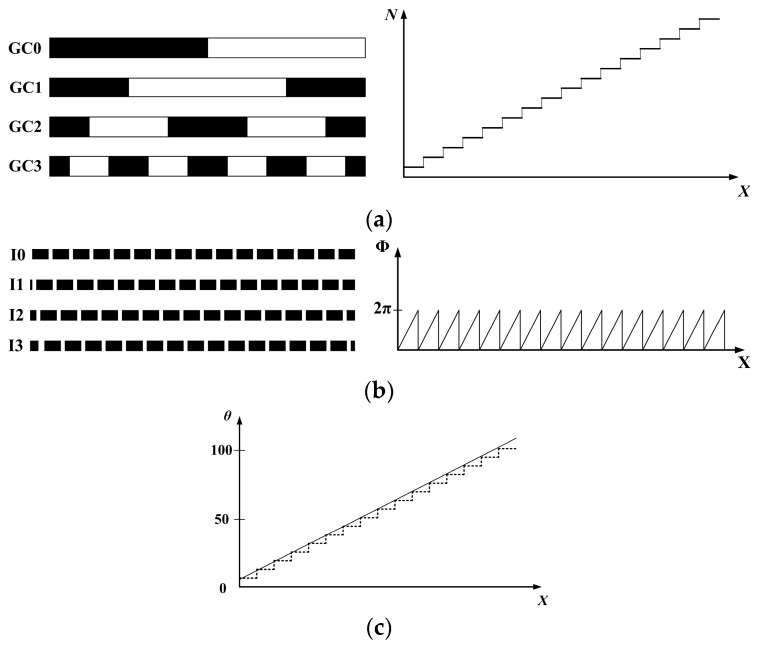
Patterns encoding methods: (**a**) Four-bit gray-code; (**b**) Four-step phase-shifting; and, (**c**) absolute phase by combining four-bit gray-code and four-step phase-shifting.

The projected patterns in the structured light measurement system are always encoded by a combination of gray code and four-step phase shifting [36,37]. As shown in Figure 3a, the 4-bit gray code encodes 2^4^ sets of subdivision by projecting four successive gray code stripes. Although the gray code method can encode the pixel accuracy, and the encoding process does not consider the spatial neighborhood, the spatial resolution of this encoding method is low because the limitation is caused by the number of projecting stripe patterns. Furthermore, the four-step phase shifting has high spatial resolution in every projection. However, the drawback of the phase shifting method is the ambiguity problem that occurs when determining the signal periods in the camera images, which is decided by the periodic nature of the patterns. When gray code methods and phase shifting methods are integrated, their positive features can be additive, and the measurement of discontinuous surfaces with fine details can even be obtained.

The four-step phase shifting algorithm has been extensively applied in optical measurement because of their speed and accuracy. The four fringe images can be represented as follows:

(4) $$I_{i}(x,\;y)=I^{\prime }(x,\;y)+I^{\prime \prime }(x,\;y)\cos[\phi (x,\;y)+2i\pi /4]$$

where $I^{\prime }(x,\;y)$ is the average intensity, $I^{\prime \prime }(x,\;y)$ is the intensity modulation, i=1, 2, 3, 4, and ϕ(x,y) are the phase, which is solved as follows:

(5) $$\phi (x,\;y)=\arctan[\frac{I_{4}(x,\;y)-I_{2}(x,\;y)}{I_{1}(x,\;y)-I_{3}(x,\;y)}]$$

where ϕ(x,y) is the wrapped phase, as shown in Figure 3b, which lies between 0 and 2πrad. However, the absolute phase is useful for the following work as the phase unwrapping detects the 2π discontinuities and removes them by adding or subtracting multiples of 2π point by point. In other words, the phase unwrapping finds integer number k so that:

(6) Φ(x, y)=ϕ(x, y)+2kπ

where Φ(x, y) is the absolute phase, and k is the number of stripe. When the phase shifting period coincides with the gray code edges, as shown in Figure 3, the phase shifting works in the subdivision, as defined by the gray code encoding method, and the absolute phase is distributed linearly and spatially continuously over the each subdivision area. Thus, all of the pixels in the camera image are tracked by their absolute phases.

### 2.3. Digital Binary Defocusing Technique

The digital binary defocusing technique was used to create computer generated binary structured fringe patterns, and the defocused projector blurred them into sinusoidal structured fringe patterns. Mathematically, the defocusing effect can be simplified to a convolution operation, and can be written as follows:

(7) $$I(x,y)=I_{b}(x,y)⊗Psf(x,y)$$

where, ⊗ represents convolution, $I_{b}(x,y)$ indicates the inputted binary fringe patterns, I(x,y) denotes the outputted smooth fringe patterns, and Psf(x,y) is the points spread function, determined by a pupil function of the optical system f(u,v).

(8) $$Psf(x,y)={\left|\frac{1}{2\pi }\int \int_{-\infty }^{+\infty }f(u,v)e^{i(xu+yv)}dudv\right|}^{2}$$

**Figure 4:**
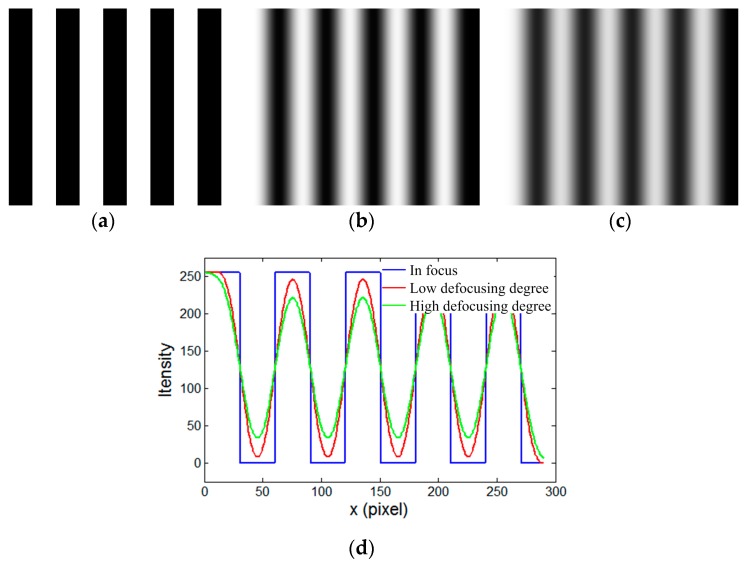
Simulation of the binary defocusing technique. (**a**) Binary structured pattern; (**b**) sinusoidal fringe pattern with low defocusing degree; (**c**) sinusoidal fringe pattern with high defocusing degree; and, (**d**) cross-section.

Simply, Psf(x,y) can be approximated by a circular Gaussian function [38,39],

(9) $$Psf(x,y)=G(x,y)=\frac{1}{2\pi \sigma^{2}}\exp(-\frac{1}{2\sigma^{2}}(x^{2}+y^{2}))$$

where the standard deviation σ is proportional to the defocusing degrees. In addition, the defocused optical system is equivalent to a spatial two-dimensional (2D) low-pass filter. As shown in Figure 4, an unaltered binary fringe pattern was simulated to generate the sinusoidal fringe pattern with increasing σ. Figure 4a shows the initial binary structured fringe pattern. Figure 4b,c represent the generated sinusoidal fringe patterns with a low defocusing degree and high defocusing degree, respectively. Figure 4d shows the cross-sections. As seen in Figure 4, when the defocusing degree increased, the binary structures became decreasingly clear, and the sinusoidal structures became increasingly obvious, which unfortunately results in a drastic fall in the intensity amplitude. To solve this problem, a pulse width modulation (PWM) technique was applied to high-quality sinusoidal patterns [40,41,42]. In addition, the dithering technique was proposed for wider binary pattern generation [43]. However, to obtain high-quality sinusoidal patterns with high fringe intensity amplitude, it is difficult to select a proper defocusing degree. Besides, more importantly, when the defocusing degree increased, the phase of defocusing fringe patterns was invariant [29].

## 3. Calibration Principle and Process

### 3.1. Camera Calibration

**Figure 5:**
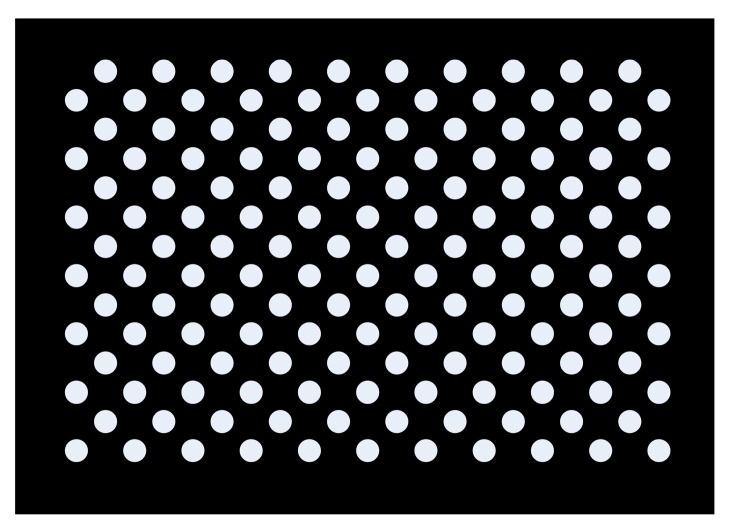
Design of the calibration board.

Essentially, the purpose of the camera calibration procedure is to obtain the intrinsic and extrinsic parameters of the camera, based on the reference data, which is composed of the 3D points on the calibration board and the 2D points on the CCD. In this research, Zhang’s method [7] was used to estimate the intrinsic parameters. Instead of using a checkerboard as calibration target, we used a flat black board with 7 × 21 arrays of white circles for calibration, as shown in Figure 5, and the centers of the circles were extracted as feature points. The calibration board was placed in different positions and orientations (poses), and 15 images were obtained to estimate the intrinsic parameters of the camera. This procedure was implemented by the OpenCV camera calibration toolbox. Notably, a typical radial and tangential distortion model was considered and the distortion was corrected for the camera calibration.

### 3.2. Out-of-Focus Projector Calibration

Generally, a projector can be regarded as an inverse camera, because it projects images rather than capturing them. If the images of the calibration points from the view of the projector are available, the projector can be calibrated as a camera, so that establishing the mapping relationship between the 2D points on the DMD of the projector and the 2D points on the CCD of the camera could realize our goal. Moreover, defocusing the projector complicates the calibration procedure. This occurs because the model for calibrating the projector briefly follows the model for the camera calibration, and since the pinhole camera model always asks for the camera to be focused, an out-of-focus projector does not directly follow this requirement. In addition, most of the projectors have noticeable distortions outside their focus plane (projection plane) [19]. In this Section, a novel calibration model for a defocusing projector is introduced, as well as a solution to the problem of calibrating an out-of-focus projector.

#### 3.2.1. Out-of-Focus Projector Model

In the literature [26], two methods perform the binary defocusing technique. The first method is that the projector shoots on the different planes with a fixed the focal length. The second method is that the projector is in different focus distances, and the plane is at a fixed location. The first defocusing degree of each method represents that the projector is in focus. To study the influence of the binary defocusing technique on the calibration results of the projector, we calibrated a projector under different defocusing degrees with the proposed method in [29], and the amount of the defocusing degree increases from 1 to 5. Table 1 shows the calibration results of the projector with different defocusing degrees, with the defocusing degree increasing from 1 to 5, caused by the first method mentioned above. Table 2 shows the calibration results of the projector with different defocusing degrees with the defocusing degree increasing from 1 to 5, caused by the second method.

**Figure 6:**
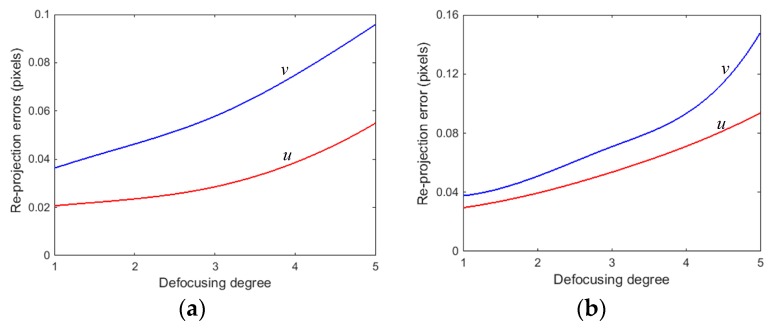
Re-projection errors under different defocusing degrees: (**a**) obtaining the different defocusing degrees using the first method; and, (**b**) obtaining the different defocusing degrees using the second method.

From Table 1, the stability of the focal length and the principal point were poor under different defocusing degrees using the first method. The maximum change of $f_{u}$ and $f_{v}$ reached 50 pixels. The maximum change of $u_{0}$ and $v_{0}$ reached 24 pixels and 40 pixels, respectively. In addition, the re-projection errors in u and v directions rose significantly as the defocusing degree of the projector increased. This is also seen in Figure 6a. Similarly, we applied the different defocusing degrees by using the second method, and the same statistical calibration results are shown in Table 2 and Figure 6b. The results show that the calibration results of the second defocusing method are basically the same as those of the first defocusing method. Therefore, all of the parameters are varying when the projector shoots on the different planes, or when the projector is in different focus distances. This is because the parameters are influenced by the projector defocusing, and there are mutually constrained relationships between parameters in the projector calibration process. 

**Figure 7:**
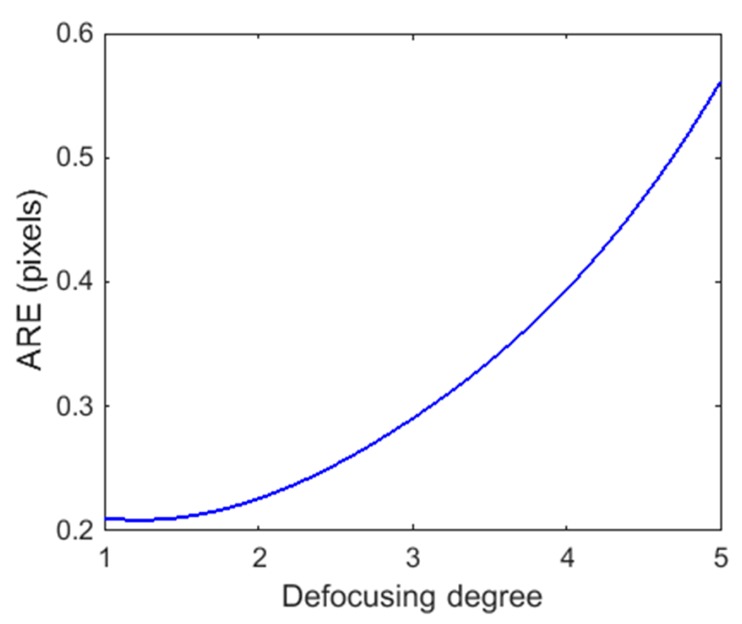
The ARE of a projector under different defocusing degrees.

In addition, we found that the distortion coefficients had varied with the increasing amount of the defocusing degree from the above experiments. Moreover, it had mentioned that most of the projectors have noticeable distortions outside their focus plane in [19]. Therefore, we decided to determine the lens distortion of the out-of-focus projector under the different defocusing degrees, which was easy to be described by the average residual error (ARE) value. The ARE was defined as follows:

(10) $$ARE=\frac{1}{n}\sum_{i=1}^{n}\sqrt{{\left(x_{i}-x_{i}^{id}\right)}^{2}+{\left(y_{i}-y_{i}^{id}\right)}^{2}}$$

where ${\left(x_{i},y_{i}\right)}_{i}$ are the actual computed image coordinates on the image plane with calibration parameters, ${\left(x^{id},y^{id}\right)}_{i}$ are the (extracted) ideal image coordinates. Table 3 shows the statistic of the ARE of an out-of-focus projector under different defocusing degrees. Figure 7 shows the varying of ARE under different defocusing degrees.

From Table 3 and Figure 7, the ARE of the projector rose as the defocusing degree of the projector increased. This indicated that the projector always had noticeable distortions outside their focus plane, and it was very useful for our further discussion.

**Figure 8:**
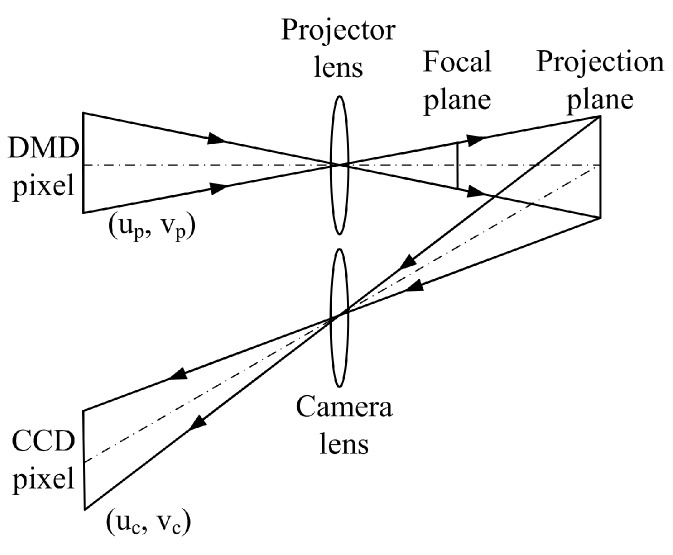
Model of a structured light system with an out-of-focus projector.

The structured light system model with an out-of-focus projector is shown in Figure 8. To generate the sinusoidal fringes from binary patterns, the projector was substantially out of focus. From the statistical calibration results of a defocused projector shown in Figure 6, the re-projection errors gradually increased as the projector defocusing increased. This occurred because we directly used the pinhole camera model to calibrate the out-of-focus projector. However, the pinhole camera model requires the camera on the focal plane. When the amount of defocusing is minimal, the re-projection errors of the projector are few, such as when using defocusing degrees 1 and 2. Conversely, if the projector is on a high defocusing degree, then the pinhole camera model is not suitable, and the re-projection errors of the projector become increasingly larger, such as when using defocusing degrees 4 and 5. Therefore, using a pinhole camera model to calibrate an out-of-focus projector will introduce errors into the calibration results. In order to improve the calibration precision, this paper proposes an out-of-focus projector calibration method that is based on nonlinear optimization with the lens distortion correction on the projection plane.

**Figure 9:**
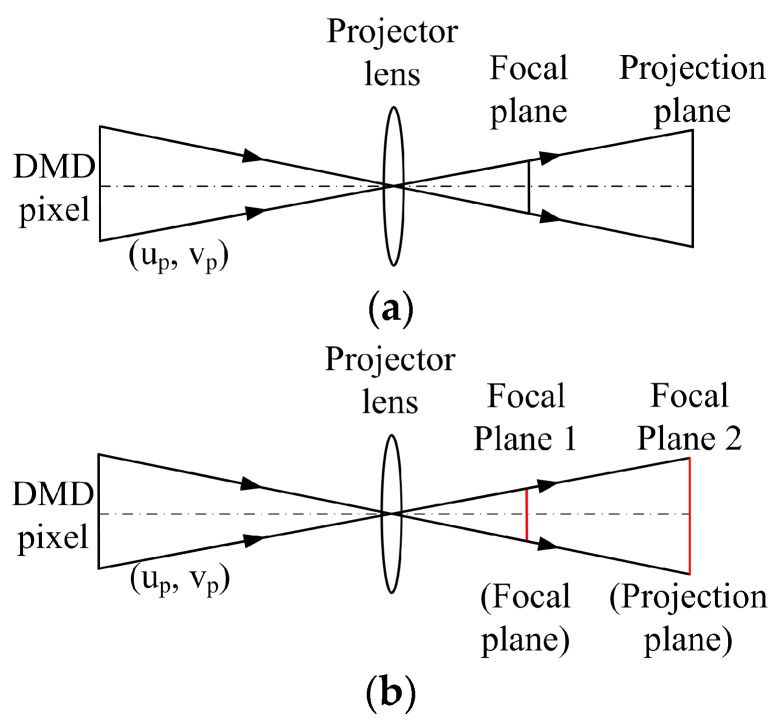
Model of an out-of-focus projector: (**a**) original model; and, (**b**) double the focal plane model.

As introduced in Section 2.1, the pinhole camera model describes a camera with intrinsic parameters, such as focal length, principle point, and pixel skew factor; and, extrinsic parameters, such as rotation matrix and translation vector. A model of an out-of-focus projector is shown in Figure 9a. For convenience of expression, the parameters or points related to the focal plane are represented by the subscript “1”, whereas the parameters or points that are related to the projection plane are represented by the subscript “2”. If the defocusing degree of the projector is determined, the unique correspondence between focal plane and projection plane will be decided. Then, the calibration plane can be moved to the focal plane of the projector. The projector can be calibrated as an inverse camera by the pinhole camera model. This process can be described as follows:

(11) $$s{\tilde{p}}_{p1}=A_{p1}[R_{p1}\;,\;T_{p1}]{\tilde{P}}_{w}$$

(12) $$A_{p}{}_{1}=\left[\begin{array}{ccc} f_{x1} & \gamma_{1} & u_{0}{}_{1}\\ 0 & f_{y1} & v_{0}{}_{1}\\ 0 & 0 & 1 \end{array}\right]$$

(13) $$\{\begin{array}{c} u_{p1}^{\prime }=u_{p1}+\delta_{up1}=u_{p1}+k_{1}u_{r1}r^{2}+k_{2}u_{r1}r^{4}+p_{1}(3u_{r1}^{2}+v_{r1}^{2})+2p_{2}u_{r1}v_{r1}\\ v_{p1}^{\prime }=v_{p1}+\delta_{vp1}=v_{p1}+k_{1}v_{r1}r^{2}+k_{2}v_{r1}r^{4}+p_{2}(u_{r1}^{2}+3v_{r1}^{2})+2p_{1}u_{r1}v_{r1} \end{array}$$

The $A_{p1},R_{p1},T_{p1}$ were selected as the initial values of the final out-of-focus projector calibration.

To obtain accurate calibration results, the lens distortion was considered. In this paper, we used a radial and tangential distortion model [30,31]. Figure 9a shows a model of an out-of-focus projector. Obviously, a distance exists between the focal plane and the projection plane. As the projector defocusing degree increases, then this distance also increases. The measurement plane (projection plane) will move away from the focal plane. Additionally, a studied showed that most projectors have noticeable distortions outside their focus plane [19]. Therefore, we considered the lens distortion on the projection plane as the initial value of the final projector calibration. However, the pinhole camera model should be used on the focal plane. To calculate the lens distortion on the projection plane using the pinhole camera model, the defocusing projector should be adjusted so it is focused on the projection plane. This plane is noted by focal plane 2, as shown in Figure 9b. Then, the projector can be calibrated as a standard inverse camera, and the lens distortion on the projection plane $\delta_{up2}$ and $\delta_{vp2}$ can be obtained. Similarly, the calibration model of the projector on the projection plane can be described as follows:

(14) $$s{\tilde{p}}_{p2}=A_{p2}[R_{p2}\;,\;T_{p2}]{\tilde{P}}_{w}$$

(15) $$A_{p}{}_{2}=\left[\begin{array}{ccc} f_{x2} & \gamma_{2} & u_{0}{}_{2}\\ 0 & f_{y2} & v_{0}{}_{2}\\ 0 & 0 & 1 \end{array}\right]$$

(16) $$\{\begin{array}{c} u_{p2}^{\prime }=u_{p2}+\delta_{up2}=u_{p2}+k_{1}u_{r2}r^{2}+k_{2}u_{r2}r^{4}+p_{1}(3u_{r2}^{2}+v_{r2}^{2})+2p_{2}u_{r2}v_{r2}\\ v_{p2}^{\prime }=v_{p2}+\delta_{vp2}=v_{p2}+k_{1}v_{r2}r^{2}+k_{2}v_{r2}r^{4}+p_{2}(u_{r2}^{2}+3v_{r2}^{2})+2p_{1}u_{r2}v_{r2} \end{array}$$

The $\delta_{up2},\delta_{vp2}$ are selected as the initial values of the final out-of-focus projector calibration.

Here, two instructions are required. First, when we calculated the intrinsic and extrinsic parameters on the focal plane, the projector condition was the same as in the final defocusing state. The parameters were close to the real value. Therefore, the intrinsic and extrinsic parameters on the focal plane were selected as the initial value of the nonlinear optimization. Secondly, with an increase in the projector defocusing degree, the measurement plane (projection plane) moved away from the focal plane. As a prior study showed that most projectors have noticeable distortions outside their focus plane [19]. We considered the lens distortion on the projection plane (focal plane 2) as the initial value of the final out-of-focus projector calibration. Finally, the initial value of an out-of-focus projector with a nonlinear optimization algorithm can be described as follows:

(17) $$A_{pp0}=A_{p1}=\left[\begin{array}{ccc} f_{x1} & \gamma_{1} & u_{0}{}_{1}\\ 0 & f_{y1} & v_{0}{}_{1}\\ 0 & 0 & 1 \end{array}\right]$$

(18) $$\left[R_{pp0},T_{pp0}]=[R_{p1},T_{p1}\right]$$

(19) $$\{\begin{array}{c} \delta_{upp0}=\delta_{up2}=k_{1}u_{r2}r^{2}+k_{2}u_{r2}r^{4}+p_{1}(3u_{r2}^{2}+v_{r2}^{2})+2p_{2}u_{r2}v_{r2}\\ \delta_{vpp0}=\delta_{vp2}=k_{1}v_{r2}r^{2}+k_{2}v_{r2}r^{4}+p_{2}(u_{r2}^{2}+3v_{r2}^{2})+2p_{1}u_{r2}v_{r2} \end{array}$$

Based on the re-projection mathematical model with distortion, the final accuracy parameters of the out-of-focus projector were obtained with a nonlinear optimization algorithm. The objective function was to minimize the sum of the re-projection error of all the reference points onto the projector image plane. This can be described as:

(20) $$[A_{pp},R_{pp},T_{pp},K_{pp}]=\arg\min{\sum_{i=1}^{N}\sum_{j=1}^{M}\left\|p_{ij}-F(A_{p1},R_{p1},T_{p1},K_{p2},P_{ij})\right\|}^{2}$$

where $\left[A_{pp},R_{pp},T_{pp},K_{pp}\right]$ are the final accuracy parameters of the out-of-focus projector; N is number of reference points; M is the number of images for projector calibration; $A_{p1}$ is the intrinsic parameter matrix on the focal plane 1; $R_{p1}$ and $T_{p1}$ represent the extrinsic parameters on the focal plane 1; $K_{p2}$ represents the distortion coefficients on the focal plane 2; $p_{ij}$ is the point coordinate on the image plane; and, F is the function representing the re-projection process of the projector. $P_{ij}$ shows the space coordinate of a calibration point. It is a nonlinear optimization problem that can be solved using the Levenberg-Marquardt method [44]. In addition, a good initial value can be provided by coarsely calibrating the parameters of the out-of-focus projector on the focal plane and projection plane, respectively.

#### 3.2.2. Phase-Domain Invariant Mapping

**Figure 10:**
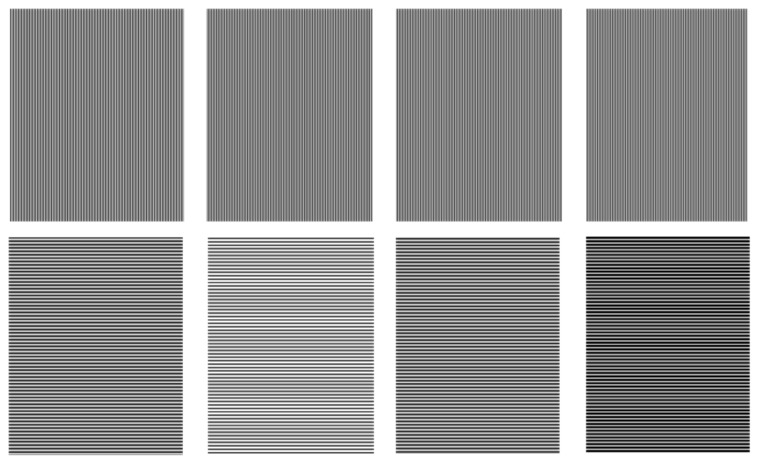
The four generated images in the first row are the vertical phase shifting fringe images $I_{V_{1}},I_{V_{2}},I_{V_{3}}$, and $I_{V_{4}}$, the corresponding horizontal phase shifting fringe images $I_{H_{1}},I_{H_{2}},I_{H_{3}}$, and $I_{H_{4}}$ are shown in the second row.

**Figure 11:**
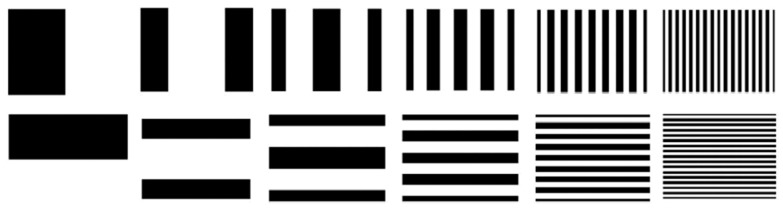
Generated gray-code patterns.

To improve the calibration accuracy of an out-of-focus projector, a unique one-to-one mapping between the pixels on the projector DMD and the pixels on the camera CCD should be virtually established in the phase domain using the phase shifting algorithm. The mapped projector images were generated, as proposed in [17], and the basic mapping principle can be described as follows. If the vertical structured light patterns, which are encoded with a combination of gray code and phase shifting, are projected onto the calibration board, and the camera captures the patterns images, the absolute phase $\Phi_{V}(u_{c},v_{c})$ can be retrieved for all of the camera pixels with Equations (5) and (6). Following the same process, if horizontal structured light patterns are projected, the absolute phase $\Phi_{H}(u_{c},v_{c})$ is extracted. In addition, the accuracy of the phase mapping is decided by high-quality phase generation, which is dependent on fringe width and the number of fringes in the phase shift method. In this paper, the four-step phase shifting algorithm was used, and the sinusoidal fringe pattern period was 16 pixels, as shown in Figure 10. Hence, six gray code patterns should be used, which have the same period as the sinusoidal fringe pattern, as shown in Figure 11. These vertical and horizontal absolute phases can be used to construct the mapping between the pixels on the DMD and CCD, as follows:

(21) $$\{\begin{array}{c} \Phi_{V}(u_{c},v_{c})=\Phi_{V}(v_{p})\\ \Phi_{H}(u_{c},v_{c})=\Phi_{H}(u_{p}) \end{array}$$

**Figure 12:**
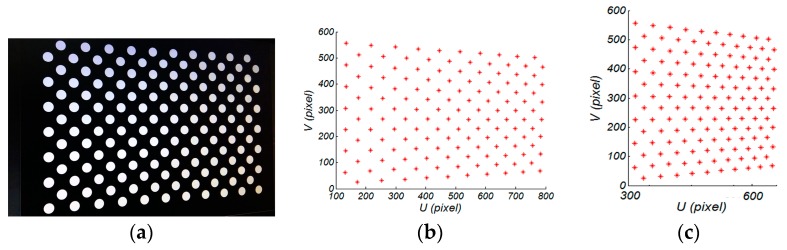
Example of the extracted correspondences circle centers for the camera and projector. (**a**) example of one calibration pose; (**b**) camera; and, (**c**) projector.

Equation (21) provides a pixel-pixel mapping from CCD to DMD; Figure 12 illustrates an example of the extracted correspondences for a single translation.

### 3.3. Out-of-Focus ProjectorCalibration Process

The camera can be calibrated with an OpenCV camera calibration toolbox. If the image coordinates of the calibration points on the DMD are obtained by Phase-Domain invariant mapping, an out-of-focus projector can be calibrated using the abovementioned approach. Specifically, the calibration process requires the following major steps:

Step 1: Image capture. The calibration board was placed on the preset location, and a white paper was stuck on the surface of the calibration board. A set of horizontal and vertical gray code patterns was projected onto the calibration board. These fringe images were captured by the camera. Similarly, the pattern images were captured by projecting a sequence of horizontal and vertical four-step phase shifting fringes. After, the white paper was removed, and the calibration board image was captured. For each pose, a total of 21 images were recorded, which were used to recover the absolute phase using the combination of gray code and the four-step phase shifting algorithm, introduced in Section 2.2.
Step 2: Camera calibration and determining the location of the circle centers on the DMD. The camera calibration method recommended in Section 3.1 was used. For each calibration pose, the horizontal and vertical absolute phase maps $\Phi_{H}(u_{c},v_{c}),\Phi_{V}(u_{c},v_{c})$ were recovered. A unique point-to-point mapping between CCD and DMD was determined as follows:
  (22) $$\{\begin{array}{c} u_{p}=\frac{\Phi_{V}(u_{c},v_{c})}{2\pi }T_{V}\\ v_{p}=\frac{\Phi_{H}(u_{c},v_{c})}{2\pi }T_{H} \end{array}$$
  where $T_{V},T_{H}$ is the four-step phase shifting patterns period in the vertical and horizontal directions, respectively. In this paper, $T_{V}=T_{H}=16$ pixels. Using Equation (22), the phase value was converted into projector pixels. Furthermore, we assigned the sub-pixel absolute phases, as obtained by the bilinear interpolation of the absolute phases of its four adjacent pixels, because of the sub-pixel circle center detection algorithm for the camera image. For high accuracy camera circle centers, the standard OpenCV toolbox was used. Figure 12 shows an example of the extracted correspondences for a single translation.
Step 3: Calculate the initial values of the intrinsic and extrinsic parameters on the focal plane (focal plane 1). To find approximate parameters, 15 different positions and orientation (poses) images were captured within the scheme measurement volume for the projector calibration. If the reference calibration data on focal plane 1 for the projector were extracted from Step 2, the coarse intrinsic and extrinsic parameters of an out-of-focus projector can be estimated using the same software algorithms for camera calibration on focal plane 1, which was described in Section 3.2.
Step 4: Compute the initial value of the lens distortion on the projection plane. According to the results of our previous experiments in Section 3.2.1, the lens distortion varies with an increasing defocusing degree. To find the approximate parameters, the lens distortion on the projection plane was considered as the initial value of the lens distortion for an out-of-focus projector. In this process, the projector was adjusted to focus on the projection plane, which was called focal plane 2. With the calibration points on focal plane 2 and their corresponding image points on the DMD, the lens distortion on the projection plane was obtained using the pinhole camera model.
Step 5: Compute the precise calibration parameters of the out-of-focus projector by using a nonlinear optimization algorithm. All of the parameters were solved by minimizing the following cost function, as outlined in Equation (20).

## 4. Experiment and Discussion

**Figure 13:**
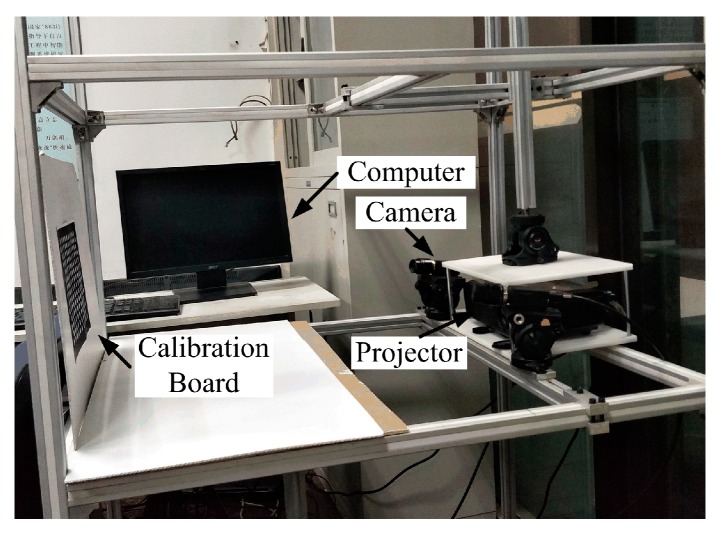
Experimental system.

To verify the validity of the proposed calibration method for an out-of-focus projector, in-laboratory experiments were completed. The experimental system is shown in Figure 13: it was composed of a DLP projector (model: OptomaDN322) with 1024 × 768 pixel resolution, and a camera (model: Point Grey GX-FW-28S5C/M-C) with a 12 mm focal length lens (model: KOWA LM12JC5M2). The camera pixel size was 4.54 µm × 4.54 µm with highest resolution of 1600 × 1200 pixels. Here, a calibration board with 7 × 21 arrays of white circles printed on a flat black board was used, and a calibration volume of 200 × 150 × 200 mm was attained. Then, the system calibration followed the method described in Section 3. To evaluate the performance of the proposed calibration method, the system was also calibrated with calibration method in [29]. In addition, the calibration images were generally contaminated by noise during image acquisition, image capturing or image transmission. In order to improve the accuracy of the feature point extraction and phase calculation, the original images in the experiment were preprocessed by an image de-noising method that was based on weighted regularized least-square algorithm [45], which can effectively eliminate the image noise and keep edge information without blurring image edge. This can help reduce the noise impact and improve the calibration results.

**Figure 14:**
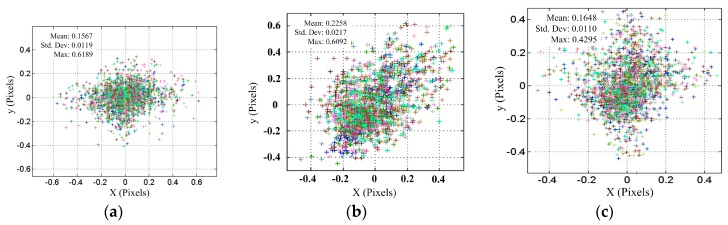
Re-projection errors of the calibration points on the image planes: (**a**) camera; (**b**) out-of-focus projector using the conventional method in [29]; and, (**c**) out-of-focal projector using the proposed method.

Table 4 shows the calibration system parameters using both our proposed method and the conventional method in [29] under defocusing degree 2, as mentioned in Table 1. As was shown, the camera parameters were almost the same for both methods, whereas the out-of-focus projector parameters were obviously different. This is because the calibration process for the projector in [29] was not reliable due to the influence of the projector defocusing. In addition, the re-projection errors of the calibration data on the camera and out-of-focus projector image planes are shown in Figure 14a–c. As shown in Figure 14a, when the re-projection errors of the calibration data on the camera image plane are 0.1567 ± 0.0119 pixels, the re-projection errors of the calibration data for the out-of-focus projector using the method in [29] are 0.2258 ± 0.0217 pixels, as shown in Figure 14b. To compare the effect of both methods, the re-projection errors of the camera remained unchanged, and the re-projection errors of the calibration data for the out-of-focus projector using the proposed method decreased to 0.1648 ± 0.0110 pixels, as shown in Figure 14c, which is a reduction of 27.01% when compared to the method in [29].

**Figure 15:**
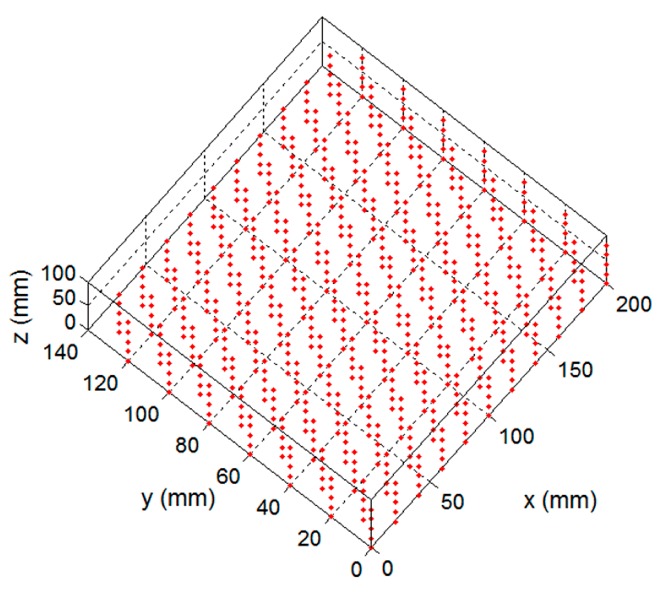
The measured distances in a 420 × 150 × 100 mm volume.

**Figure 16:**
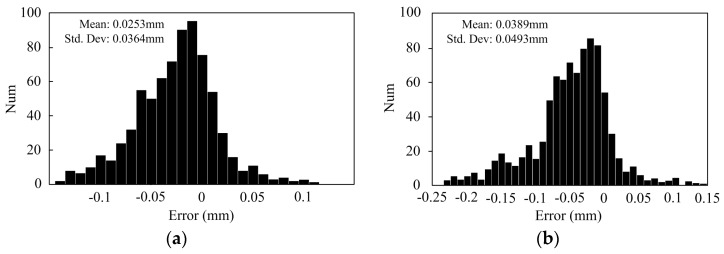
Histogram of the measurement error of the 20 mm distances by: (**a**) the proposed method; and (**b**) the method in [29].

To evaluate the performance of the proposed calibration method, the standard distances between two adjacent points on the calibration board in the x and y directions were measured using the two methods. In addition, the standard distances were obtained by moving the calibration board 20 mm in the parallel direction within the volume of 200 × 140 × 100 mm, and the total number of the 1295 distances was measured. Figure 15 shows the measured distances within the 200 × 140 × 100 mm volume. Figure 16 shows the histogram of the distribution of the distance measurement error. The measuring error was 0.0253 ± 0.0364 mm for our proposed calibration method, as shown in Figure 16a, the measuring error was 0.0389 ± 0.0493 mm for the calibration method in [29], shown in Figure 16b. The measurement accuracy and the uncertainty were improved by 34.96% and 26.17%, respectively.

**Figure 17:**
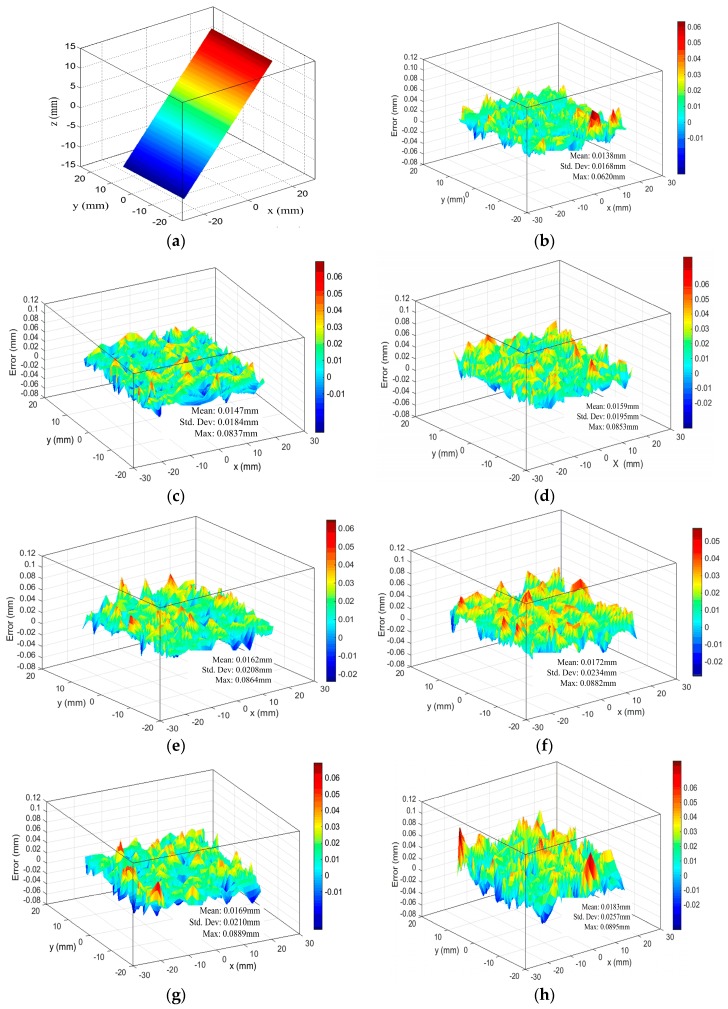

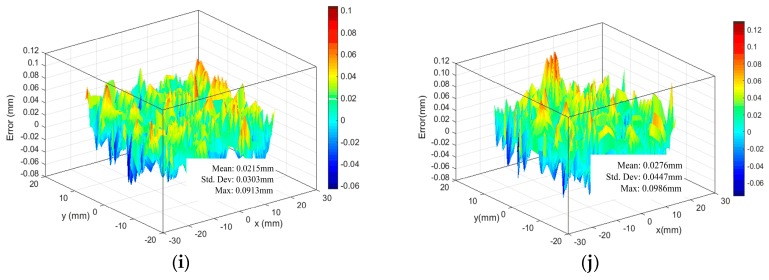
Measurement results and error of plane under different defocusing degrees: (**a**) fitting plane; (**b**) measurement error of the plane under defocusing degree 1 (projector in focus); (**c**–**f**) measurement error of the plane under defocusing degree 2–5 by our proposed calibration method; (**g**–**j**) corresponding the measurement error of the plane under defocusing degree 2–5 by the calibration method in [29].

**Figure 18:**
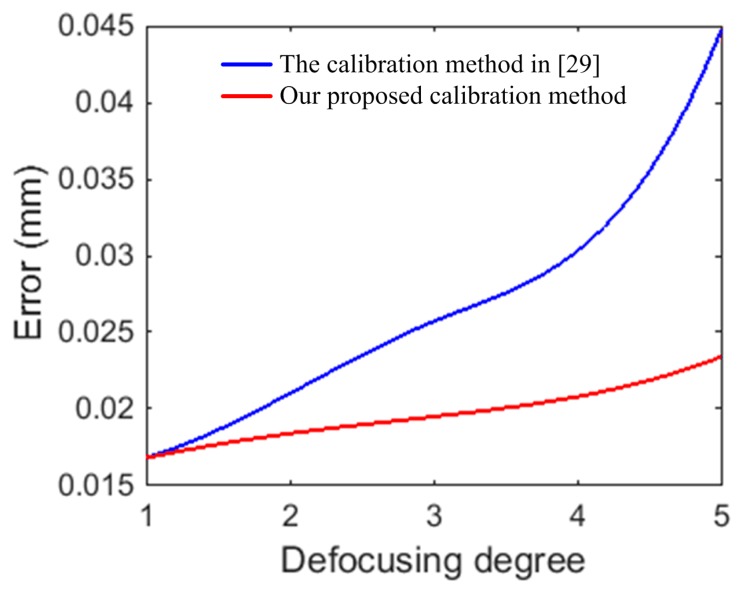
Measurement error of plane under different defocusing degrees using our proposed calibration method and the calibration method in [29].

To further test the results of our proposed calibration method, a planar board and an aluminum alloy hemisphere were measured by defocusing the camera-projector system under different defocusing degrees listed in Table 1. The measurement results of the planar board under the five defocusing degrees are shown in Figure 17. The measurement error of the board is defined as the distance between the measuring point and the fitting plane. To determine the measurement errors of the board, the board was also measured using a coordinate measuring machine (CMM) with a precision of 0.0019 mm. Table 5 presents the statistics of the measurement results of the board with five different defocusing degrees. The board fitting residuals of the CMM’s measurement data were 0.0065 ± 0.0085 mm and the maximum was less than 0.0264 mm. Figure 17a,b show the fitting plane and the fitting residuals of the plane with the projector in focus, under defocusing degree 1, respectively. Additionally, it is important to note that our calibration method is the same as the method in [29], under defocusing degree 1. So, there is only one set of measurement results. Figure 17c–f show the measurement results under defocusing degrees 2 to 5 using our proposed calibration method. Similarly, the measurement results under defocusing degrees 2 to 5 using the calibration method in [29] are shown in Figure 17g–j. When the defocusing degree is minimal, such as defocusing degrees 2 and 3, the fitting residuals were similar between our proposed calibration method and the calibration method in [29]. The fitting residuals using our calibration method were 0.0147 ± 0.0184 mm and 0.0159 ± 0.0195 mm, and 0.0169 ± 0.0210 mm and 0.0183 ± 0.0257 mm, using the calibration method in [29], respectively. However, as the defocusing degree increased to defocusing degrees 4 and 5, the differences between the measurement results were obvious. Especially for defocusing degree 5, using our proposed calibration method, the fitting residual was 0.0172 ± 0.0234 mm. Using the calibration method in [29], the fitting residual reached 0.0276 ± 0.0447 mm. Figure 18 shows the fitting error varying with the different defocusing degrees using both our proposed calibration method and the calibration method in [29]. From Figure 18, the change of the fitting error is not obvious for different defocusing degrees using our proposed calibration method. Nevertheless, the fitting error increased rapidly using the calibration method in [29]. Because our proposed calibration method considers the influence of defocusing on the calibration results.

**Figure 19:**
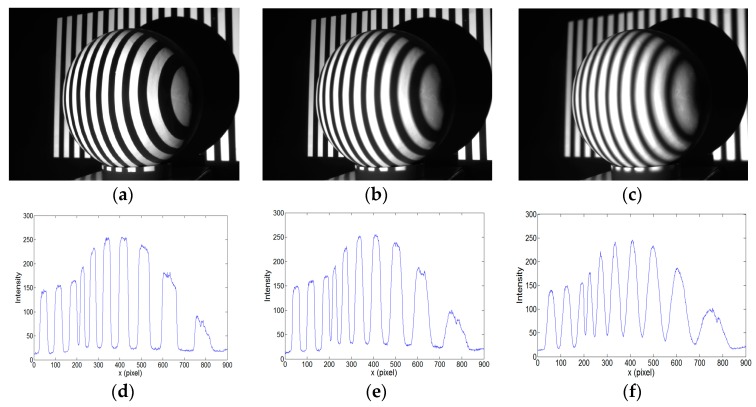
Illustration of three different defocusing degrees: (**a**) a captured fringe image under defocusing degree 1 (projector in focus); (**b**) a captured fringe image under defocusing degree 2 (projector slightly defocused); (**c**) a captured fringe image under defocusing degree 5 (projector very defocused); and, (**d**–**f**) Corresponding cross sections of the intensity of (**a**–**c**).

**Figure 20:**
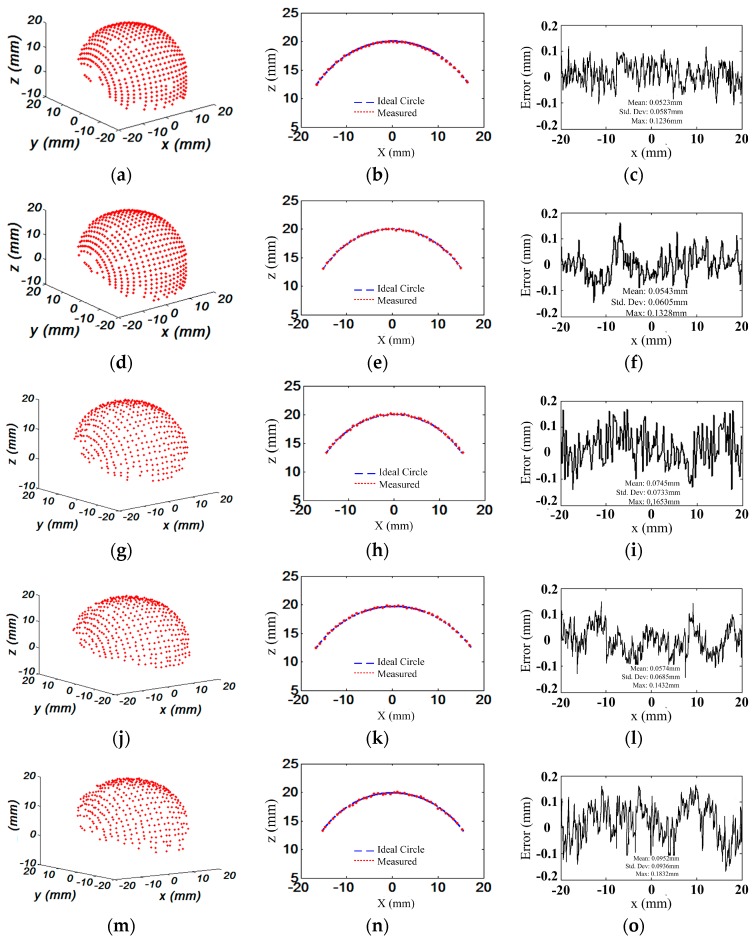
Measurement results and error of a hemisphere surface under different defocusing degrees: (**a**) fitting hemisphere measured under defocusing degree 1 (projector in focus); (**b**) cross section of the measurement result and the ideal circle; (**c**) error estimated in x direction; (**d**–**f**) and (**j**–**l**) correspond to figures (**a**–**c**) measured using our proposed calibration method under defocusing degrees 2 and 5; and, (**g**–**i**) and (**m**–**o**) correspond to figures (**a**–**c**) measured using the calibration method in [29] under defocusing degrees 2 and 5.

An aluminum alloy hemisphere was also measured using the defocusing camera-projector system for three different defocusing degrees: defocusing degrees 1, 2, and 5. The captured fringe images for the three defocusing degrees and their cross sections of intensity are shown in Figure 19. The measurement and statistics results are shown in Figure 20 and Table 6, respectively. The measurement results under defocusing degree 1 (projector in focus) are shown in Figure 20a–c. Figure 20a shows the reconstructed 3D surface. To evaluate the accuracy of measurement, we obtained a cross section of the hemisphere and fitted it with an ideal circle. Figure 20b shows the overlay of the ideal circle and the measured data points. The error between these two curves is shown in Figure 20c. Figure 20d–f and Figure 20j–l show the measurement results under defocusing degrees 2 and 5 using our proposed calibration method, respectively. Correspondingly, Figure 20g–i and Figure 20m–o show the measurement results for defocusing degrees 2 and 5 using the calibration method in [29], respectively. 

**Figure 21:**
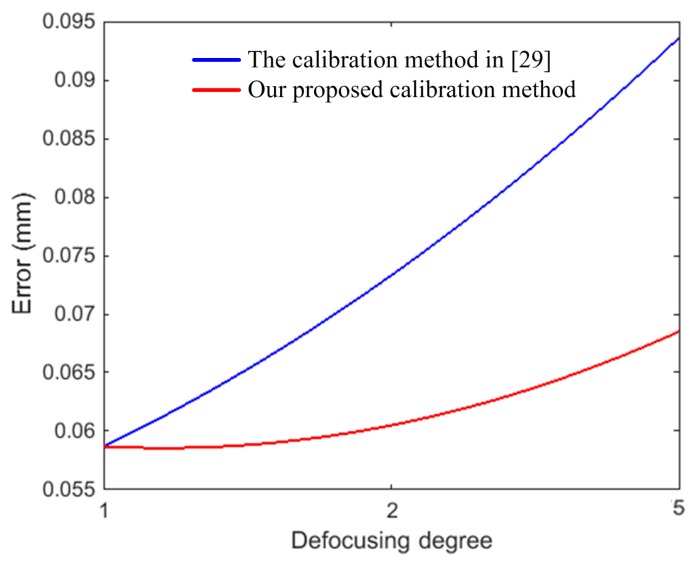
Measurement error of the hemisphere under different defocusing degrees using our proposed calibration method and the calibration method in [29].

To evaluate our proposed calibration method, the hemisphere was also measured by a CMM with a precision of 0.0019 mm. The fitting radius of the CMM’s measurement data was 20.0230 mm, and the hemisphere fitting residuals was 0.0204 ± 0.0473 mm. The fitting radius of defocusing camera-projector system that was calibrated by our proposed calibration method was 19.9542 mm, which had a deviation of 0.0688 mm from the fitting radius of the CMM’s measurement data. The hemisphere fitting residuals was 0.0543 ± 0.0605 mm for defocusing degree 2. Furthermore, with the same experimental conditions, the hemisphere was also measured by the defocusing camera-projector system with calibration method proposed in [29]. The fitting radius with the point data of the hemisphere was 19.9358 mm, which had a deviation of 0.0872 mm from the CMM’s fitting radius, and the hemisphere fitting residuals was 0.0745 ± 0.0733 mm. For defocusing degree 5, the hemisphere fitting residual was 0.0574 ± 0.0685 mm using our proposed calibration method, and 0.0952 ± 0.0936 mm using the calibration method in [29]. Similarly, the fitting error varying with the different defocusing degrees using our proposed calibration method and the calibration method in [29] is shown in Figure 21. From Figure 21, the change of the fitting error was not obvious for different defocusing degrees using our proposed calibration method. Nevertheless, the fitting error increased rapidly when using the calibration method in [29]. Thus, the measurement results using the camera-projector system with our proposed projector calibration method were better than the method in [29]. All of the experimental results verified that the camera and out-of-focus projector system attain satisfactory accuracy using our proposed projector calibration method.

## 5. Conclusions

This paper proposes an accurate and systematic calibration method to calibrate an out-of-focus projector in a structured light system using a binary defocusing technique. To achieve high accuracy, the calibration method includes two parts. Firstly, good initial values are provided by coarsely calibrating the parameters of the out-of-focus projector on the focal plane and projection plane. Secondly, the final accuracy parameters of the out-of-focus projector are obtained using a nonlinear optimization algorithm, based on the re-projection mathematical model with distortion. Specifically, a polynomial distortion representation on the projection plane, and not the focal plane in which the high order radial and tangential lens distortion are considered, was used to reduce the residuals of the projection distortion. In addition, the calibration points in the camera image plane were mapped to the projector according the phase of the planar projection. The experimental results showed that satisfactory calibration accuracy was achieved using our proposed method, regardless of the defocusing amount. Of course, this method is not without its limitations. When compared to the traditional calibration method, the computing was somewhat longer. 

## Figures and Tables

**Table 1:** Calibration results of a projector under different defocusing degrees when using the first method.

Defocusing Degree	$f_{u}f_{v}$	$u_{o}v_{0}$	$K=\left[\begin{array}{cc} k_{1} & p_{1}\\ k_{2} & p_{2} \end{array}\right]$		Re-Projection Errors	
					u	v
1	2033.15992	481.85752	−0.00501	0.00741	0.02071	0.03627
	2029.32333	794.04516	0.10553	−0.00781		
2	2066.07579	480.66343	0.00025	0.00075	0.02356	0.04631
	2060.38058	817.00819	−0.02750	−0.00774		
3	2066.02355	482.08093	−0.01202	−0.00303	0.02856	0.05781
	2061.98782	824.49272	−0.01985	−0.00817		
4	2083.24450	457.53174	−0.03166	−0.00456	0.03862	0.07487
	2079.93614	834.40594	0.08715	−0.01328		
5	2082.66646	458.68461	−0.09894	−0.01726	0.05492	0.09577
	2090.35224	801.22181	0.13975	−0.01243		

**Table 2:** Calibration results of a projector under different defocusing degrees when using the second method.

Defocusing Degree	$f_{u}f_{v}$	$u_{o}v_{0}$	$K=\left[\begin{array}{cc} k_{1} & p_{1}\\ k_{2} & p_{2} \end{array}\right]$		Re-Projection Errors	
					u	v
1	2065.84461	486.73708	0.00832	−0.00052	0.02950	0.03762
	2068.06661	815.66697	−0.03886	−0.00749		
2	2074.79015	503.27888	0.00111	0.00496	0.03941	0.05078
	2074.45555	824.02444	0.03516	−0.00402		
3	2131.24929	526.11764	0.00240	−0.00229	0.05352	0.07076
	2135.41564	799.98280	0.03484	−0.00229		
4	2165.81230	541.55585	−0.02157	−0.00543	0.07101	0.09316
	2166.67073	794.56998	0.17737	0.00042		
5	2173.40017	531.46318	0.01313	−0.00125	0.09358	0.14790
	2172.43898	810.09141	0.10122	−0.00151		

**Table 3:** Average residual error (ARE) of a projector under different defocusing degrees (unit: pixel).

Statistic	Defocusing Degree				
	1	2	3	4	5
ARE	0.21043	0.22585	0.29015	0.39374	0.56108

**Table 4:** Calibration results of the camera and out-of-focus projector.

Method	Device	$f_{u}f_{v}$	$u_{0}v_{0}$	$K=\left[\begin{array}{cc} k_{1} & p_{1}\\ k_{2} & p_{2} \end{array}\right]$		R		T	
Proposed method	Camera	2708.93985	684.18114	−0.01640	0.00698	0.94231 −0.01042 −0.25359	−0.00342 0.99926 −0.00936	0.23016 0.00653 0.73162	−389.37651 179. 73663 229.32452
		2732.74604	740.39548	0.03143	−0.00944				
	Projector	2065.25354	461.4964	−0.06638	−0.00567				
		2061.88752	798.62552	0.02323	−0.00562				
The method in [29]	Camera	2708.93865	684.16035	−0.01640	0.00698	0.94242 −0.01038 −0.25338	0.00364 0.99952 −0.00929	0.23024 0.00681 0.73139	−389.35619 180.0085 230.02781
		2732.74653	740.39749	0.03143	−0.00944				
	Projector	2066.07579	480.66343	0.00025	0.00075				
		2060.38058	817.00819	−0.02750	−0.00774				

**Table 5:** Statistics of the measurement results of plane (unit: mm).

Statistic	Defocusing Degree	Mean	SD	Max.
Plane by CMM	Null	0.0065	0.0085	0.0264
Plane by camera-projector system with our proposed projector calibration method	1	0.0138	0.0168	0.0620
	2	0.0147	0.0184	0.0837
	3	0.0159	0.0195	0.0853
	4	0.0162	0.0208	0.0864
	5	0.0172	0.0234	0.0882
Plane by camera-projector system with the proposed projector calibration method in [29]	1	0.0138	0.0168	0.0620
	2	0.0169	0.0210	0.0889
	3	0.0183	0.0257	0.0895
	4	0.0215	0.0303	0.0913
	5	0.0276	0.0447	0.0986

**Table 6:** Statistics of the measurement results of hemisphere (unit: mm).

Statistic	Defocusing Degree	Fitting Radius	Mean	SD	Max.
Hemisphere by CMM	Null	20.0230	0.0204	0.0473	0.1165
Hemisphere by camera-projector system with our proposed projector calibration method	1	19.9745	0.0523	0.0587	0.1236
	2	19.9542	0.0543	0.0605	0.1328
	5	19.9537	0.0574	0.0685	0.1432
Hemisphere by camera-projector system with the proposed projector calibration method in [29]	1	19.9745	0.0523	0.0587	0.1236
	2	19.9358	0.0745	0.0733	0.1653
	5	19.9108	0.0952	0.0936	0.1832
